# Umbelliferone instability during an analysis involving its extraction process

**DOI:** 10.1007/s00706-018-2188-9

**Published:** 2018-06-28

**Authors:** Andrzej L. Dawidowicz, Katarzyna Bernacik, Rafał Typek

**Affiliations:** Faculty of Chemistry, Maria Curie Sklodowska University, Pl. Maria Curie Sklodowska 3, 20-031 Lublin, Poland

**Keywords:** Extraction, High-pressure liquid chromatography, Mass spectroscopy, Umbelliferone, Coumarins, Umbelliferone transformation

## Abstract

**Abstract:**

Umbelliferone (7-hydroxycoumarin) is one of the most popular compounds of the coumarins family. This compound receives the attention of scientists due to its diverse bioactivities in a number of applications in various therapeutic fields. An interesting aspect of umbelliferone is its structural lability. The enzymatic degradation process of umbelliferone to its hydroxylated (esculetin), glucosylated (skimmin), and methylated (herniarin) derivatives is already known from the literature. In this paper, we describe the possibility of umbelliferone transformation to other derivatives. We found that eight compounds were formed from umbelliferone during its simulated extraction under reflux performed in different conditions (different heating times and solvents used). Six of them (4,7-dihydroxy-3,4-dihydro-2*H*-chromen-2-one, 3,7-dihydroxy-3,4-dihydro-2*H*-chromen-2-one, methyl (2*E*)-3-(2,4-dihydroxyphenyl)prop-2-enoate, ethyl (2*E*)-3-(2,4-dihydroxyphenyl)prop-2-enoate, (2*E*)-3-[2-(acetyloxy)-4-hydroxyphenyl]prop-2-enoic acid, (2*E*)-3-(2-amino-4-hydroxyphenyl)prop-2-enoic acid) have not been reported yet. Some of these compounds were also identified in extracts of plant materials containing umbelliferone—chamomile flower and cinnamon bark. Compound separation was carried out using the HPLC apparatus. All compounds were identified based on their MS fragmentation paths. The presented results are useful for food producers and consumers, as umbelliferone transformation products can be formed during food product storage, their preparation or processing.

**Graphical abstract:**

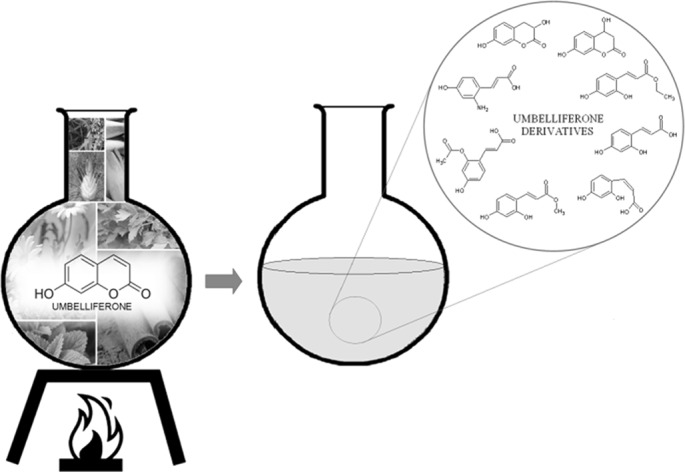

**Electronic supplementary material:**

The online version of this article (10.1007/s00706-018-2188-9) contains supplementary material, which is available to authorized users.

## Introduction

The increasing interest in healthy lifestyle, including healthy nutrition, is responsible for the search for plant-derived compounds with pro-health activity. Apart from popular polyphenols, the attention of food and pharmaceutical agent producers has recently been focused on coumarins, a group of biologically active plant components. In the food industry, coumarins are used as flavoring additives in wines, liqueurs, dietary supplements, and as seasonings for sauces, soups, and meats. They are also found in products containing cinnamon (e.g., chewing gum, breakfast cereals, gingerbread) and in many vegetables and fruits. It is necessary to monitor coumarin concentration, primarily in food products, due to their toxic activity when eaten in excessive quantities.

Umbelliferone, known also as 7-hydroxycoumarin, skimmetine, or hydrangine, is one of the most widespread coumarins (1,2-benzopyrones) in the plant kingdom [1–4]. This secondary plant metabolite contributes to the adaptation of plants to biotic or abiotic stress [5]. It occurs in the flowers, fruits, and roots of all higher plants, e.g., of the Apiaceae (Umbelliferae) family [6–8]. Umbelliferone receives the attention of scientists due to its diverse bioactivities in a number of applications in various therapeutic fields [9]. The most notable is its strong ability to absorb UV light [10], and hence its usage as an ingredient of sun protection cosmetics and as an optical brightener for textiles [11–13]. Like other coumarins, it also occurs in many food products.

The analysis of plant and food constituents requires the application of sample preparation methods, allowing for full isolation of the analyzed substances from the examined material [14]. For this purpose, high-temperature extraction is most frequently used in the analytical procedures of plants and foods. Studies on the structural transformations of chlorogenic acid [15–17] and rutin [18–20] showed that classical extraction under reflux in different conditions (pH, type of extractant, temperature, heating time) led to the formation of their derivatives. This study attempts to answer the question concerning umbelliferone stability during its extraction process, which is commonly used for compound isolation from plants during their analysis.

The presented experiments are justified, as umbelliferone is the parent compound for many derivatives naturally occurring in plants, which suggests its structural lability [11–13]. Moreover, their results may be useful for food producers and consumers, as umbelliferone transformation products are likely to be formed during the preparation and processing of food products. The more so that some food preparation procedures are often carried out for a long time, in high temperatures and at different pH levels influenced by plant matrix components or other constituents of the product. Hence, the need for a study of coumarin stability is essential. The presented experiments were conducted in artificial systems and then confronted with a selected natural system.

## Results and discussion

### Umbelliferone transformation during its simulated extraction under reflux

#### Qualitative analysis

**Fig. 1:**
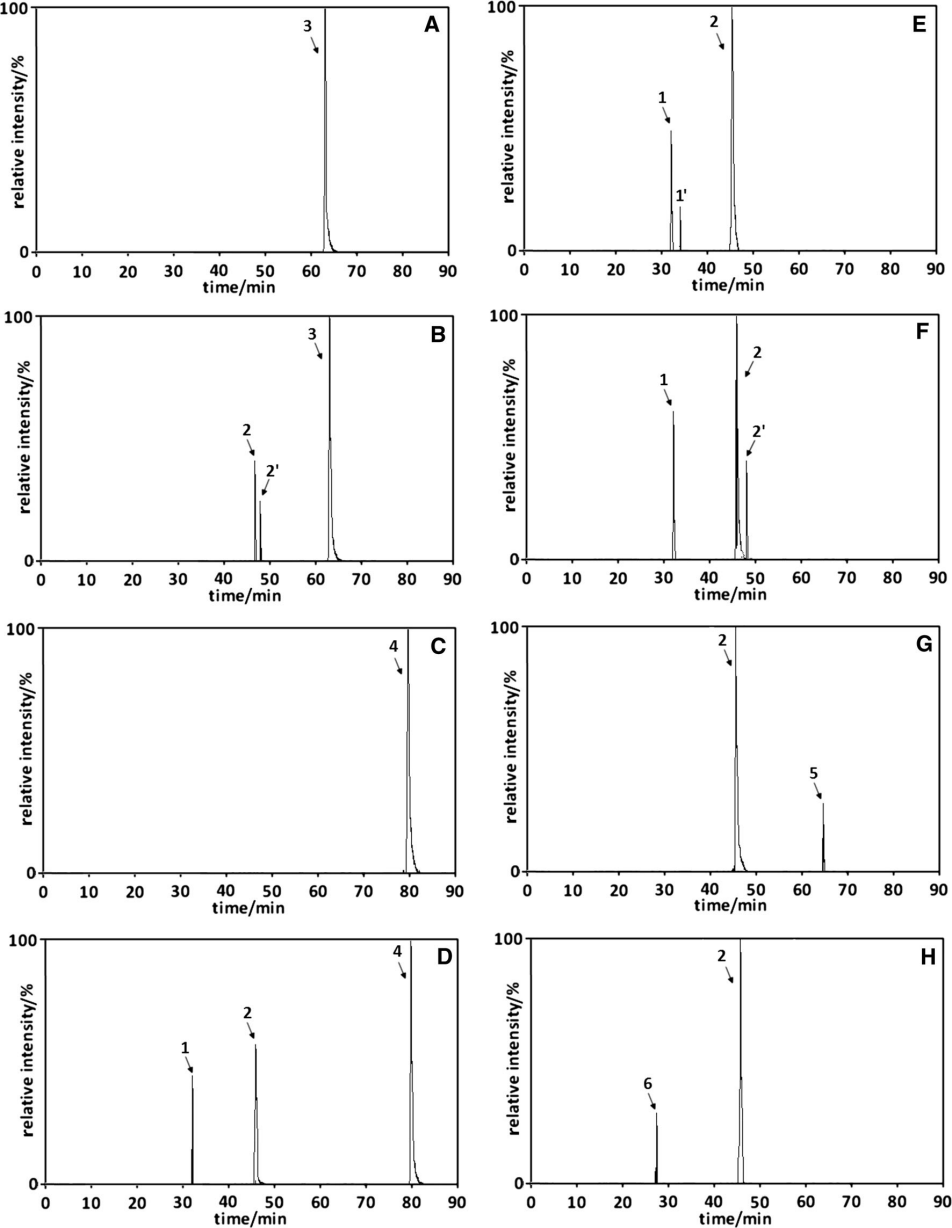
The exemplary chromatograms of umbelliferone solutions in methanol (**a**), methanol/phosphoric buffer (pH 9; 75/25 v/v; **b**), ethanol (**c**), ethanol/water (75/25 v/v; **d**), phosphoric buffer (pH 5; **e**), water (**f**), acetic buffer (pH 9; **g**), and ammonia buffer (pH 9; **h**), all heated under reflux for 2 h. Peak numbers correspond to compound numbers reported in Supplementary Scheme 1

Most formulas of plant and food analysis involve the application of liquid extraction methods such as Soxhlet extraction, percolation, maceration, and extraction under reflux [21, 22]. For the isolation of umbelliferone from plant materials, alcohol (mainly methanol or ethanol) and alcohol–water mixtures are most frequently applied as extractants [23, 24]. Water itself also draws the attention of researchers as an extraction solvent for plant components. Its possible application for the extraction process results not only from its unique properties in the superheated state, but also because it is a non-flammable, non-toxic, readily available, and environmentally friendly solvent (so-called green solvent). This is why it was also included in the experiments. Figure 1 presents the exemplary chromatograms of umbelliferone solutions in methanol (Fig. 1a), methanol/phosphoric buffer (pH 9; 75/25 v/v; Fig. 1b), ethanol (Fig. 1c), ethanol/water (75/25 v/v; Fig. 1d), phosphoric buffer (pH 5; Fig. 1e), water (Fig. 1f), acetic buffer (pH 9; Fig. 1g), and ammonia buffer (pH 9; Fig. 1h), all heated under reflux for 2 h. These solutions imitate the umbelliferone extracts obtained for this compound during its extraction under reflux by the mentioned extrahents. In these chromatograms, the umbelliferone peak was omitted and the apparent peaks represent only the products of umbelliferone transformation.

Although methanol is most often used as umbelliferone extractant for analytical purposes, however, it seems to be more convenient to start the discussion from phosphoric buffer (pH 5) extract. As shown in Fig. 1e, the chromatograms of this extract contain, besides umbelliferone, three compounds (peaks **1**, **1′**, and **2**) formed as a result of the umbelliferone transformation and/or degradation. The substances represented by peaks **1** and **1′** have been recognized as hydroxyl derivatives of umbelliferone on the basis of a few factors. The molecular weights of the compounds represented by these peaks exceed the molecular weight of umbelliferone by 18.0101 and 18.0099, respectively, and correspond to the molecular weight of water, 18.0106 Da. The introduction of the –OH group to any molecule increases its polarity, which results in a diminution of its retention in the RP chromatographic system. The retention times of the considered compounds are shorter than the retention time of umbelliferone. The MS^*n*^ spectra and mass of substances represented by peaks **1**, **1′** and of umbelliferone are very similar (see Tables 1, 2).

**Table 1:** Negative ion MS^*n*^ data for umbelliferone transformation products

Compd no. from Fig. 2	MS^1^	MS^2^		
	Base peak/*m/z*	Base peak/*m/z*	Secondary peak	
			*m/z*	Intensity/%
**1**	179.1	161.2	117.2	9.1
**1′**	179.1	161.1	117.2	10.2
**2**	179.2	135.1	–	–
**2′**	179.2	135.2	–	–
**3**	193.1	179.1	135.1	7.3
**4**	207.1	179.1	135.2	6.6
**5**	221.1	179.2	178.2 135.1	13.2 8.7
**6**	178.2	134.2	–	–

**Table 2:** HRMS data for umbelliferone transformation products

Compd no. from Fig. 2	Elemental composition [M–H]^−^	[M–H]^−^/Da		Error	
		Experimental	Calculated	/ppm	/mDa
**1**	C_9_H_7_O_4_	179.0337	179.0344	3.9	− 0.7
**1′**	C_9_H_7_O_4_	179.0336	179.0344	4.5	− 0.8
**2**	C_9_H_7_O_4_	179.0343	179.0344	0.6	− 0.1
**2′**	C_9_H_7_O_4_	179.0349	179.0344	2.8	0.5
**3**	C_10_H_9_O_4_	193.0509	193.0501	4.1	0.8
**4**	C_11_H_11_O_4_	207.0651	207.0657	2.9	− 0.6
**5**	C_11_H_9_O_5_	221.0457	221.0450	3.2	0.7
**6**	C_9_H_8_O_3_N	178.0508	178.0504	2.2	0.4

As results from the literature [9], the addition of an –OH group to carbon neighboring a phenyl group (carbon 4) is more privileged. Hence, peak **1** corresponds to umbelliferone hydroxyl derivative with the –OH group in position 4. Slightly longer retention of umbelliferone hydroxyl derivative with the –OH group in position 3 (peak **1′**) results from its somewhat greater hydrophobicity—the –OH group in position 3 is involved in the creation of hydrogen bond with the carbonyl group. For the substance represented by peak **2** (Fig. 1e), it has been recognized as an isomer *trans* of umbellic acid (2,4-dihydroxycinnamic acid). Such primary identification results from its MS^2^ spectrum, which agrees with the literature data for this compound [25] and proves the presence of a carboxyl group in the molecule (see Table 1). Significantly, the molecular weight of this compound also exceeds the molecular weight of umbelliferone by 18.0101, the consequence of the opening of lactone structure due to the reaction of umbelliferone with water.

Figure 1f presents an exemplary chromatogram of umbelliferone solutions in water, which shows that, apart from the parent substance, three additional compounds, formed in the process of the umbelliferone transformation and/or degradation, are present in the mixture. Two of them (peaks **1** and **2**) have been described above. They are: umbelliferone hydroxyl derivative with the –OH group in position 4 (peak **1**) and umbellic acid (peak **2**). The compound represented by peak **2′** can be identified as *cis*-umbellic acid. Its MS^2^ spectrum is the same as the MS^2^ spectrum for peak **2**. This primary identification is additionally confirmed by its longer retention in the RP-HPLC system than the retention of *trans*-umbellic acid. The longer retention of the *cis* form is due to the formation of an internal hydrogen bond in this compound resulting in the increase of the hydrophobic character of the molecule. Moreover, in relation to *trans*, the *cis* form is thermodynamically less stable and in consequence its concentration in the examined extract is lower.

Figure 1a presents an exemplary chromatogram of umbelliferone solution in pure methanol, which shows that, besides the parent substance, only one additional compound is formed as a result of umbelliferone transformation and/or degradation. Its molecular weight exceeds that of umbelliferone by the molecular weight of methanol. It can be identified as either the methoxyl derivative of umbelliferone or methyl ester of umbellic acid. However, its MS^2^ spectrum, similar to the MS^2^ spectrum of umbellic acid (see Table 1), indicates the second of the mentioned compounds. Two structures, *trans* and *cis*, of this compound can be formed during the transesterification process. We observed only one peak corresponding to the more thermodynamically stable *trans*-methyl ester of umbellic acid.

Figure 1c presents an exemplary chromatogram of umbelliferone solutions in pure ethanol. As in methanol, only one additional compound is formed during the heating of an ethanolic solution of umbelliferone. It has been recognized as ethyl ester of umbellic acid. Its molecular weight exceeds that of umbelliferone by the molecular weight of ethanol and its MS^2^ spectrum is similar to MS^2^ of umbellic acid (see Table 1). The retention differences of peaks **2**, **3** and **3**, **4** are similar, typically for the LC separation of homologs in gradient elution (compare Fig. 1f, a, c). This fact additionally confirms the primary identification validity of the umbellic acid esters.

An exemplary chromatogram of umbelliferone solutions in methanol/phosphoric buffer (pH 9; 75/25 v/v) is shown in Fig. 1b. Besides the parent substance, it shows the presence of three additional compounds formed as a result of umbelliferone transformation and/or degradation. They are *trans*- and *cis*-umbellic acid (peaks **2** and **2′**, respectively) and *trans*-methyl ester of umbellic acid (peak **3**). The first two compounds are characteristic products of umbelliferone transformation in pure water (see Fig. 1f), whereas the third compound appears during the heating of methanolic umbelliferone solution (see Fig. 1a). Analogous commentary can be given to Fig. 1d, showing an exemplary chromatogram of umbelliferone solutions in ethanol/water (75/25 v/v). The visible chromatographic responses correspond to umbelliferone hydroxyl derivative with the –OH group in position 4 (peak **1**), *trans*-umbellic acid (peak **2**), and *trans*-ethyl ester of umbellic acid (peak **4**).

The above discussion shows that umbelliferone transformations are pH dependent. Some of the discussed experiments were performed using phosphate buffers. The question appears about the influence of buffer type on umbelliferone transformation during its heating in buffered water solutions. It is a valid question, because the umbelliferone molecules, containing hydroxyl group, and its transformation product—umbellic acid, containing carboxyl group, are able to react with different buffer components. Figure 1g, h presents exemplary chromatograms of umbelliferone solutions in acetate buffer (pH 9) and ammonia buffer (pH 9), respectively, both heated under reflux for 2 h. Their analysis shows that besides *trans*-umbellic acid (peak **2**), both refluxed solutions contain umbellic acid derivative, acyl derivative (see peak **5** in Fig. 1g), and amino derivative (see peak **6** in Fig. 1h). The primary identification of these derivatives results from their MS^2^ spectra (see Table 1). The retention times of these components—longer in the case of more hydrophobic acyl derivative of umbellic acid, and shorter for more polar amino derivative of umbellic acid—are additional arguments for such assignment. As shown in Supplementary Scheme 1, there are two groups in umbellic acid which can be substituted by the acyl or amino groups. According to the obtained MS data, the –OH group in the *ortho*-position related to the carboxyl group undergoes substitution in the applied experimental conditions. The substitution of the –OH groups in the *para*-position should result in the formation of acyl or amino derivatives of umbelliferone itself; yet, the reaction mixtures are free of these derivatives.

It should be emphasized that NMR identification of all umbelliferone derivatives was impossible due to their small amounts in the reaction mixtures.

#### Quantitative analysis

**Fig. 2:**
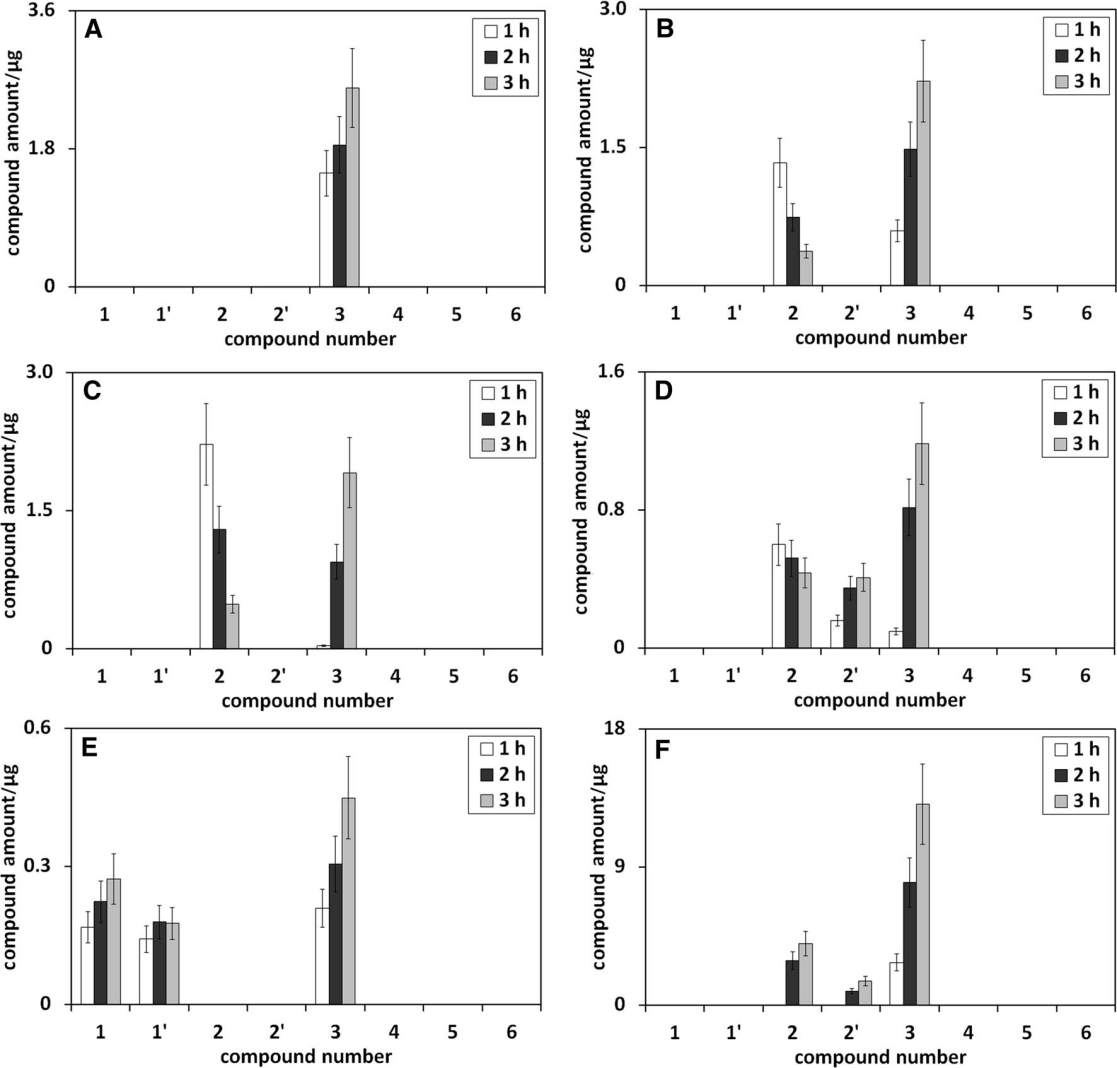
The influence of heating time on the amounts of umbelliferone derivatives in methanolic (**a**), methanol/water (75/25 v/v; **b**), methanol/water (50/50 v/v; **c**), methanol/water (25/75 v/v; **d**), methanol/phosphoric buffer (pH 5; 75/25 v/v; **e**), and methanol/phosphoric buffer (pH 9; 75/25 v/v; **f**) solution of umbelliferone, all heated under reflux. Numbers under bars correspond to compound numbers reported in Supplementary Scheme 1

Figure 2 presents the influence of heating time on the amounts of umbelliferone derivatives in methanolic (Fig. 2a), methanol/water (75/25 v/v; Fig. 2b), methanol/water (50/50 v/v; Fig. 2c), methanol/water (25/75 v/v; Fig. 2d), methanol/phosphoric buffer (pH 5; 75/25 v/v; Fig. 2e), and methanol/phosphoric buffer (pH 9; 75/25 v/v; Fig. 2f) solution of umbelliferone, all heated under reflux.

Figure 2a shows that the increase of heating time of methanolic umbelliferone solution leads to the concentration increase of *trans*-methyl ester of umbellic acid. This obvious effect results from the reaction kinetics of the transformation process of the examined compound in methanol. The same effect, the concentration increase of *trans*-methyl ester of umbellic acid with the increase of heating time, is also observed for methanol/water solutions of umbelliferone (see Fig. 2b–d). However, its intensity declines with the decrease of methanol concentration in methanol/water mixture.

For *trans*-umbellic acid, the next umbelliferone transformation product in methanol/water mixtures, an opposite effect of heating time increase on the compound concentration is seen (see bars corresponding to compound **2** in Fig. 2b–d). The observed trend can be explained by two parallel reactions: the transformation of umbelliferone to *trans*-umbellic acid and the reaction of the latter compound with methanol to *trans*-methyl ester of umbellic acid.

The third transformation product formed during the heating of neutral methanol/water solutions of umbelliferone is *cis*-umbellic acid, but only at high concentrations of water, and the increase of heating time of umbelliferone solution brings about its small concentration increase (see Fig. 2d).

The strange absence of *cis*-methyl ester of umbellic acid in the presence of *cis*-umbellic acid in heated methanol/water solutions of umbelliferone may result from low concentration of the *cis*-acid and methanol (only 25%) and/or thermal instability of *cis*-methyl ester of umbellic acid itself.

As shown from the discussion so far, the path of umbelliferone transformation is pH dependent, as hydroxyl derivatives of umbelliferone appear in its acidic solutions and umbellic acids in alkaline ones. In Fig. 2e, f, illustrating the influence of heating time on the amounts of umbelliferone derivatives in methanol/phosphoric buffer (pH 5; 75/25 v/v) and in methanol/phosphoric buffer (pH 9; 75/25 v/v) solution of umbelliferone, a small concentration growth of *cis* and *trans* form of umbelliferone hydroxyl derivative (Fig. 2e), and *cis* and *trans* form of umbellic acid (Fig. 2f) is observed as a result of the longer heating time. The influence of heating time increase on the amount of *trans*-methyl ester of umbellic acid, the third umbelliferone transformation product in buffered methanolic solution, is the same as in methanolic or methanol/water solutions of umbelliferone: its elongation leads to the increase of the amount of *trans*-methyl ester of umbellic acid.

**Fig. 3:**
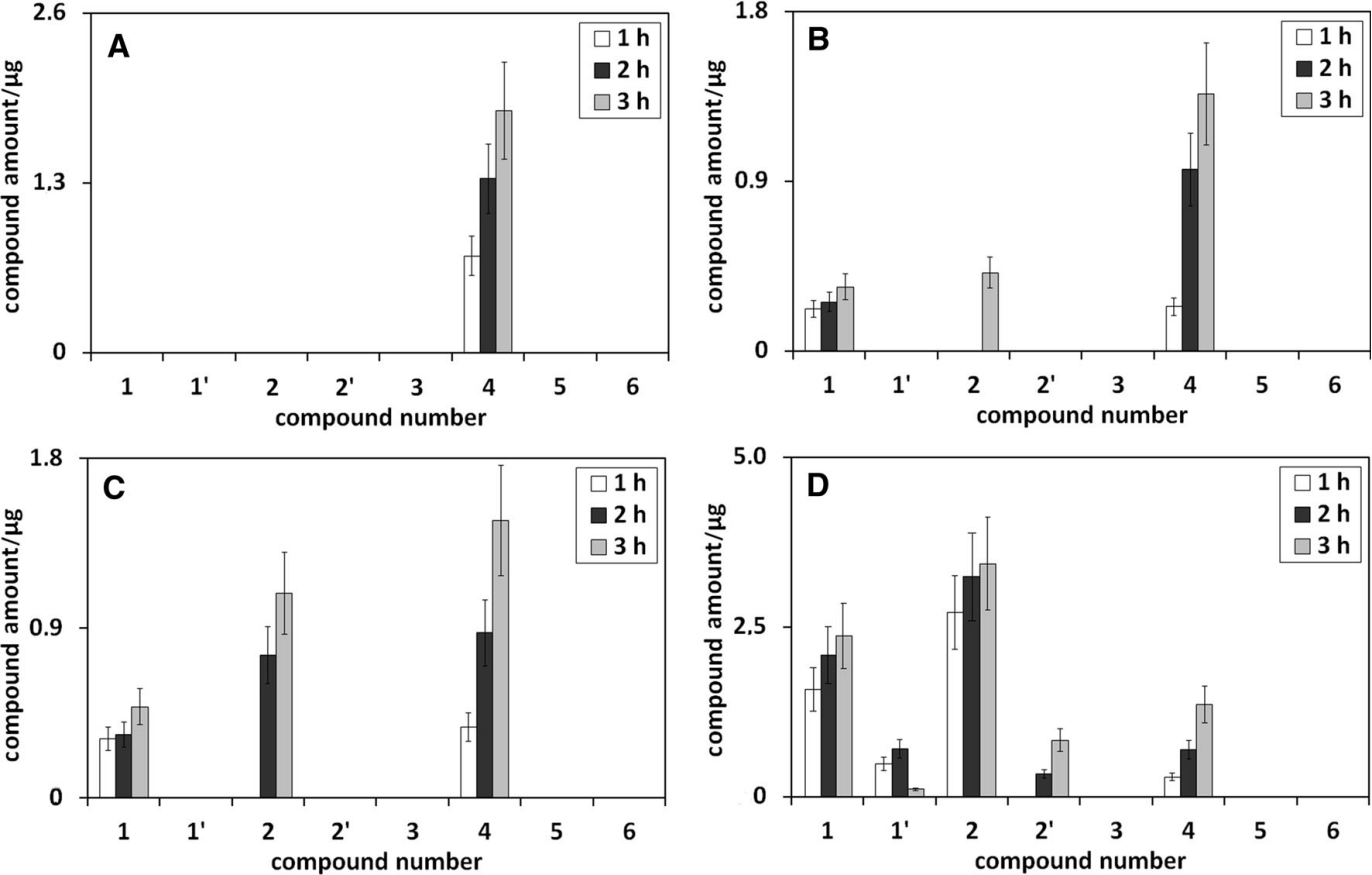
The influence of heating time on the amounts of umbelliferone derivatives in ethanolic (**a**), ethanol/water (75/25 v/v; **b**), ethanol/water (50/50 v/v; **c**), and ethanol/water (25/75 v/v; **d**) solution of umbelliferone, all heated under reflux. Numbers under bars correspond to compound numbers reported in Supplementary Scheme 1

The influence of heating time on the amount of umbelliferone derivatives in ethanolic, ethanol/water (75/25 v/v), ethanol/water (50/50 v/v), and ethanol/water (25/75 v/v) solution of umbelliferone, all heated under reflux, is presented in Fig. 3.

Following Fig. 3a, the increase of heating time of ethanolic umbelliferone solution leads to the concentration increase of *trans*-ethyl ester of umbellic acid (compound **4**). This trend results from the reaction kinetics of umbelliferone transformation in ethanol. The same shape of concentration changes of *trans*-ethyl ester of umbellic acid in longer heating time is also observed for ethanol/water solutions of umbelliferone (see Fig. 3b–d). When ethanol concentration in ethanol/water mixture decreases, the intensity of the observed trend declines.

In addition to *trans*-ethyl ester of umbellic acid, also umbelliferone hydroxyl derivatives (see **1** and **1′** in Fig. 3b–d) and umbellic acids (see **2** and **2′** in Fig. 3b–d) are formed in ethanol/water solutions of umbelliferone. For *trans*-umbellic acid (product **2**), its concentration growth with heating time increase is observed. This trend is opposite to that found for *trans*-umbellic acid in heated methanol/water solutions of umbelliferone (compare Fig. 3b–d with Fig. 2b–d). Moreover, the intensity of the trend in ethanol/water umbelliferone solutions, in relation to that in methanol/water umbelliferone solutions, is also reversed—it increases when ethanol concentration in ethanol/water umbelliferone solution decreases. This behavior can be explained by the much slower transformation of umbellic acid to its ethyl ester (product **4**) in ethanol/water umbelliferone solutions than to its methyl ester (product **3**) in methanol/water umbelliferone solutions. More acidic character of methanol favors the formation of esters.

The presence of umbelliferone hydroxyl derivatives in neutral ethanol/water umbelliferone solutions (see **1** and **1′** in Fig. 3b–d) may seem surprising; yet, it can be explained by the formation of the furan ring (lactone structure) in the cyclization process of umbellic acid. Umbelliferone transforms to umbellic acid, which is partly transformed to umbelliferone hydroxyl derivative with the –OH group in position 4 (product **1**) and eventually 3 (product **1′**), and partly to its ethyl ester. The formation of lactones from hydroxyl-aromatic acids is known from the literature [9]. As the transformation of umbellic acid to its methyl ester is much quicker than to ethyl ester, umbelliferone hydroxyl derivatives in more acidic methanolic umbelliferone solutions are not visible.

**Fig. 4:**
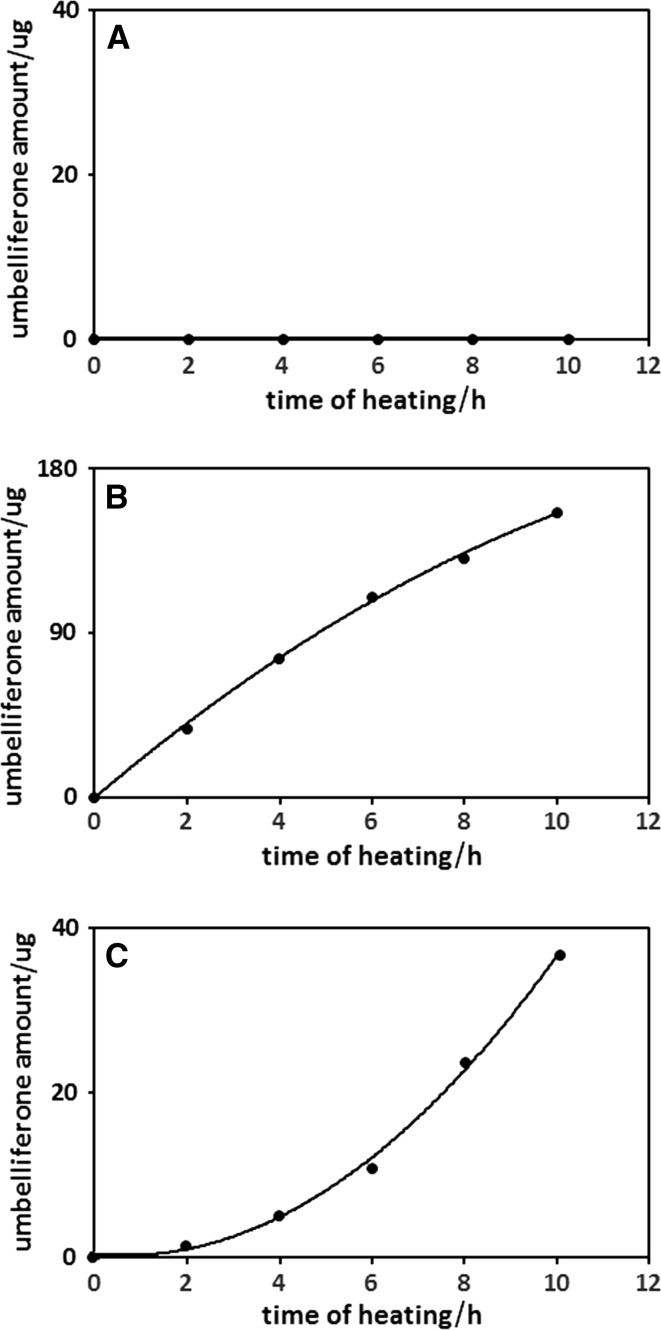
The influence of heating time on the amounts of umbelliferone in methanol/phosphoric buffer (pH 5; 75/25 v/v; **a**), methanol/phosphoric buffer (pH 9; 75/25 v/v; **b**), and ethanol/water (75/25 v/v; **c**) solution of umbellic acid, all heated under reflux. Numbers under bars correspond to compound numbers reported in Supplementary Scheme 1

To confirm the assumed hypothesis about the cyclization process of umbellic acid, it was decided to perform additional experiments, heating solutions of umbellic acid in MeOH/buffer, pH 5, MeOH/buffer, pH 9, and EtOH/water (75/25 v/v) over different periods of time. The results of this experimental series are shown in Fig. 4.

As shown in Fig. 4b, c, the neutral and alkaline reaction environment favors the formation of umbellic acid lactone (umbellic acid cyclic form). Its amount is proportional to the heating time of the umbellic acid solution. Thus, these additional experiments confirm the validity of the assumption about the possible restoration of umbelliferone. The cyclic form of umbellic acid in acidic solution does not appear (see Fig. 4a).

The umbelliferone hydroxyl derivative with the –OH group in position 3 (product **1′**) and *cis*-umbellic acid (product **2′**) are present only in ethanol/water umbelliferone solutions of low ethanol concentration (see Fig. 3d). Ambiguous trend of their concentration changes with heating time increase can be connected with their thermodynamic instability and/or with experimental error caused by their low concentration in the examined extracts.

**Fig. 5:**
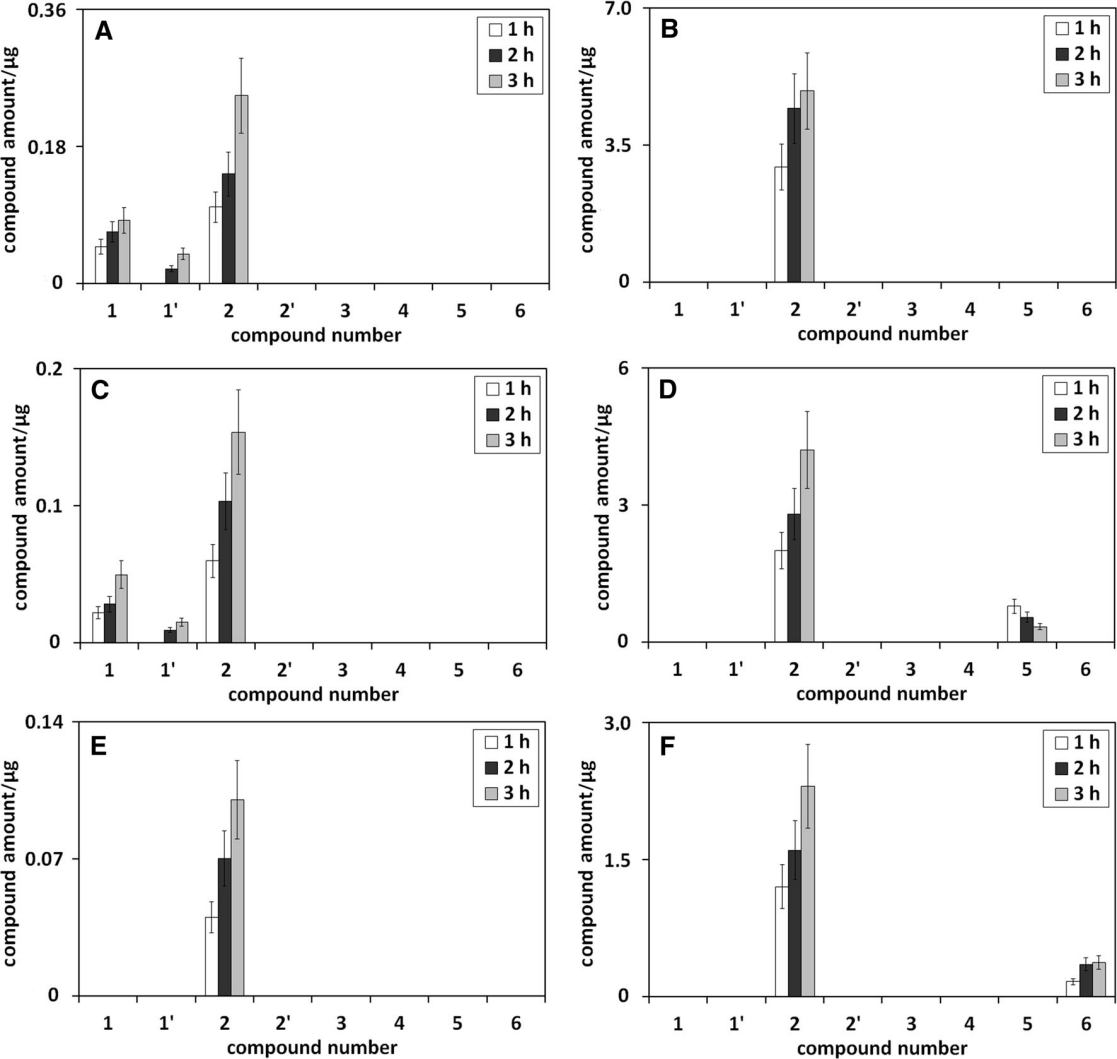
The influence of heating time on the amounts of umbelliferone derivatives in phosphoric buffer (pH 5; **a**), phosphoric buffer (pH 9; **b**), acetic buffer (pH 5; **c**), acetic buffer (pH 9; **d**), ammonia buffer (pH 5; **e**), and ammonia buffer (pH 9; **f**) solution of umbelliferone, all heated under reflux. Numbers under bars correspond to compound numbers reported in Supplementary Scheme 1

The influence of heating time on the amount of umbelliferone derivatives in phosphoric buffer (pH 5), phosphoric buffer (pH 9), acetic buffer (pH 5), acetic buffer (pH 9), ammonia buffer (pH 5), and ammonia buffer (pH 9) solution of umbelliferone, all heated under reflux, is presented in Fig. 5. It shows that the increase of heating time of umbelliferone solutions in all buffer types of pH 5 (Fig. 5a, c, e) leads to the concentration increase of *trans*-umbellic acid (transformation product **2**). The same trend is seen for umbelliferone hydroxyl derivatives, two other umbelliferone transformation products (**1** and **1′**) appearing in phosphoric buffer (pH 5) and acetic buffer (pH 5) solutions of umbelliferone. The absence of these compounds in ammonia buffer (Fig. 5e) can result from temperature destruction of this volatile buffer or can be connected with its low buffer capacity at pH 5 (the greatest capacity for this buffer is in the pH range 8.2–10.2).

The main transformation product in alkaline buffers is *trans*-umbellic acid (see Fig. 5b, d, f). Its concentration grows with the increase of alkaline umbelliferone solution heating time. The discussion of the chromatograms in Fig. 1g, h shows that not only *trans*-umbellic acid, but also acyl and amino derivative of umbellic acid (transformation product no. **5** and **6**) are formed in alkaline acetic and ammonia buffer solution of umbelliferone (see Fig. 5d, f). For the amino derivative of umbellic acid (Fig. 5f), concentration growth with heating time increase is observed. The opposite trend is seen for the acyl derivative of umbellic acid (Fig. 5f). This fact is probably connected with the low thermal stability of acyl umbelliferone derivative and/or with the thermal decomposition of acetic buffer, which additionally exhibits a low capacity at pH 9. Moreover, ester hydrolysis is promoted in an alkaline environment.

### Umbelliferone transformation during its extraction from chamomile flowers and cinnamon bark

**Fig. 6:**
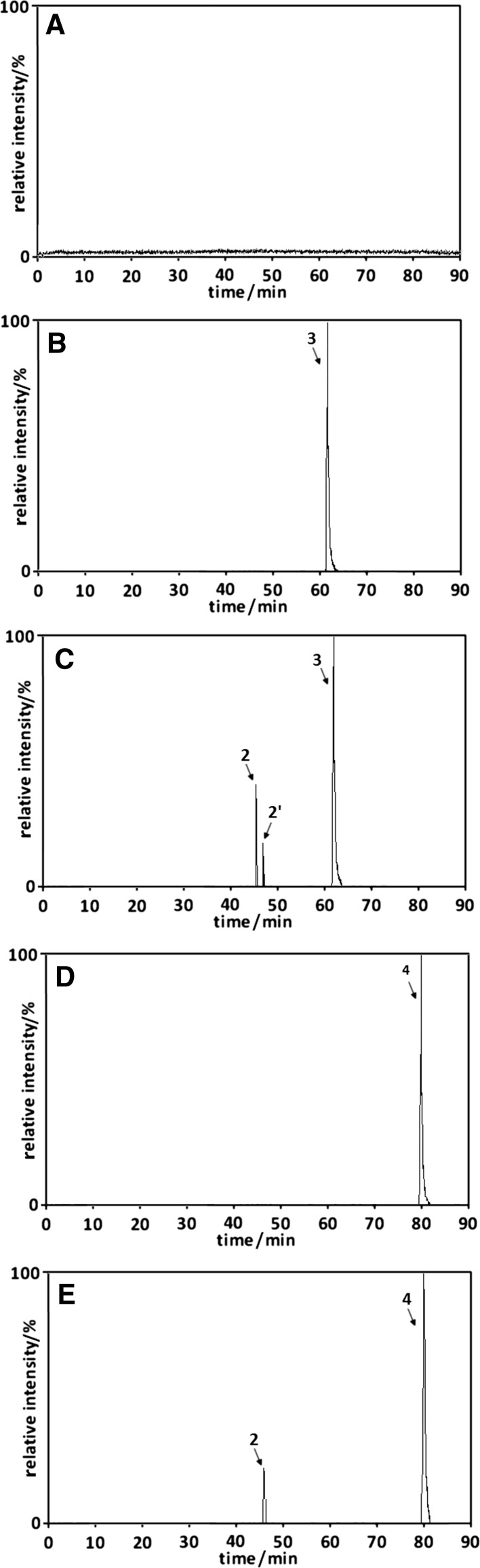
The SIM chromatograms of methanol/water (75/25 v/v) chamomile extract obtained at room temperature using exhaustive (2 h) ultrasound assisted extraction (**a**) and methanolic (**b**), methanol/phosphoric buffer (pH 9; 75/25 v/v; **c**), ethanolic (**d**), ethanol/water (75/25 v/v; **e**) extracts from chamomile, all heated under reflux for 2 h. Peak numbers correspond to compound numbers reported in Supplementary Scheme 1

Chamomile (*Chamomilla recutita* L.) is one of the most popular plants with high umbelliferone concentration. Figure 6 presents SIM chromatograms of methanolic (Fig. 6b), methanol/phosphoric buffer (pH 9; 75/25 v/v; Fig. 6c), ethanolic (Fig. 6d), ethanol/water (75/25 v/v; Fig. 6e) extracts from chamomile, all heated under reflux for 2 h, whereas Fig. 6a shows an exemplary chromatogram of methanol/water (75/25 v/v) chamomile extract obtained at room temperature using exhaustive (2 h) ultrasound-assisted extraction. It shows that none of the described umbelliferone derivatives is present in room temperature methanol/water extract (see Fig. 6a); however, the high-temperature extraction process of chamomile in extractants containing methanol or ethanol leads to umbelliferone transformation and the formation of methyl or ethyl ester of umbellic acid and umbellic acids (see Fig. 6b–e). The absence of umbelliferone hydroxyl derivative in the natural system (umbelliferone extraction from plant) is the only factor that distinguishes it from umbelliferone transformation in the artificial system. This difference is probably caused by the plant matrix components which can inhibit some umbelliferone transformation paths. Such supposition is in agreement with [19] reporting the influence of plant matrices type on the transformation of rutin during its extraction from plants.

**Fig. 7:**
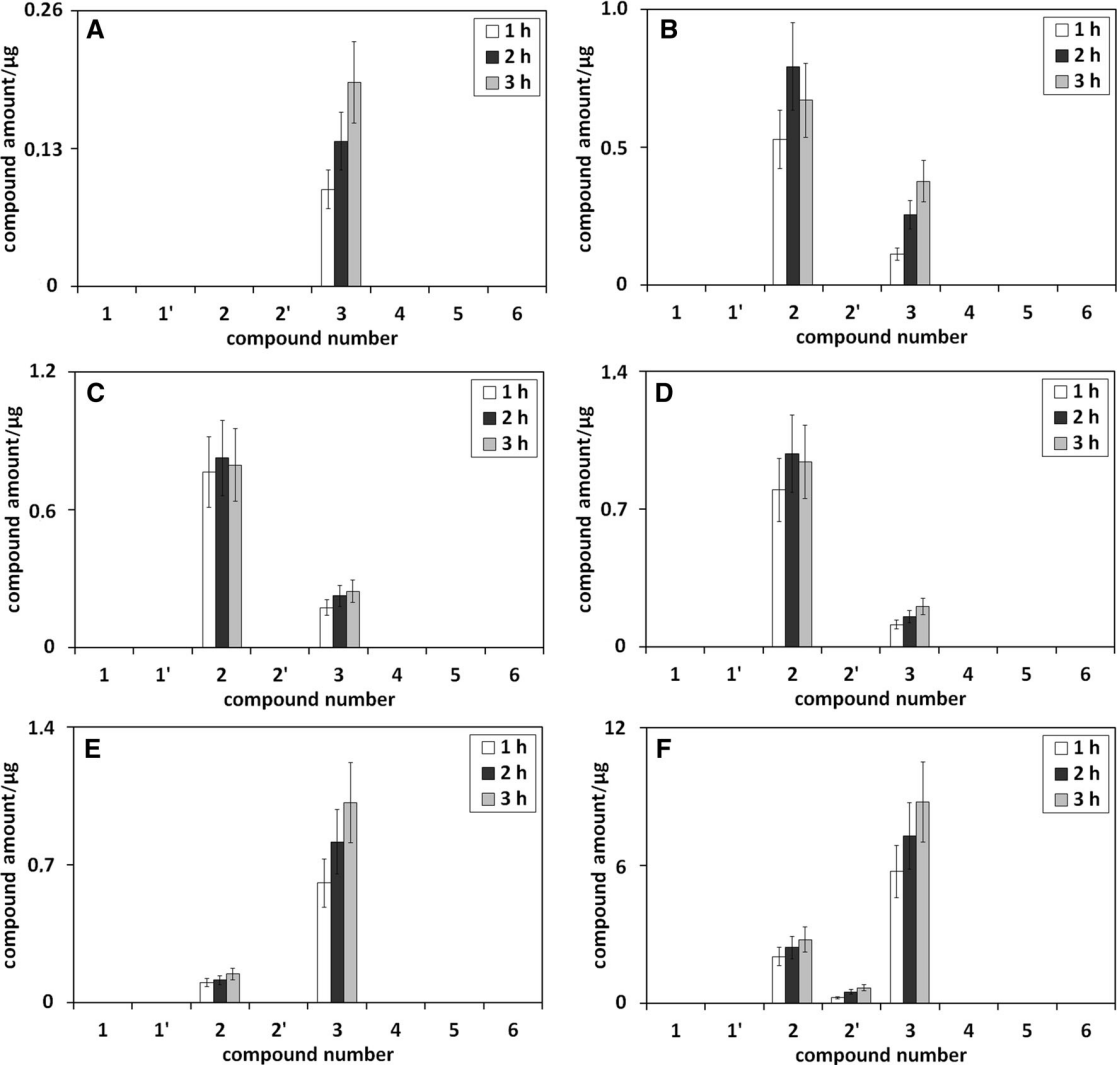
The influence of the extraction time on the amounts of umbelliferone derivatives in methanolic (**a**), methanol/water (75/25 v/v; **b**), methanol/water (50/50 v/v; **c**), methanol/water (25/75 v/v; **d**), methanol/phosphoric buffer (pH 5; 75/25 v/v; **e**), methanol/phosphoric buffer (pH 9; 75/25 v/v; **f**) extracts from chamomile, all heated under reflux. Numbers under bars correspond to compound numbers reported in Supplementary Scheme 1

**Fig. 8:**
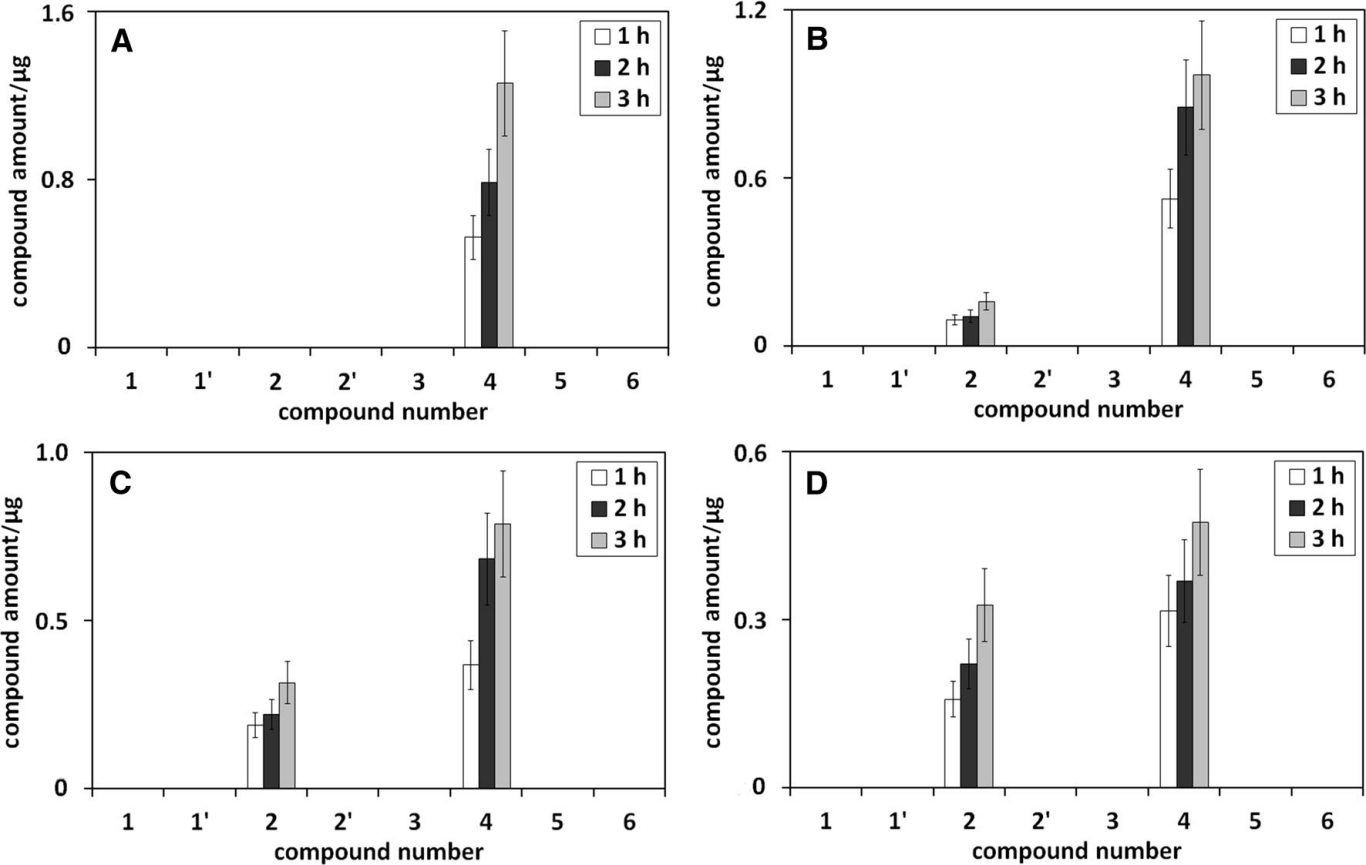
The influence of the extraction time on the amounts of umbelliferone derivatives in ethanolic (**a**), ethanol/water (75/25 v/v; **b**), ethanol/water (50/50 v/v; **c**), and ethanol/water (25/75 v/v; **d**) extracts from chamomile, all heated under reflux. Numbers under bars correspond to compound numbers reported in Supplementary Scheme 1

Figures 7 and 8 present the influence of extraction time on the amounts of umbelliferone derivatives in methanolic (Fig. 7a), methanol/water (75/25 v/v; Fig. 7b), methanol/water (50/50 v/v; Fig. 7c), methanol/water (25/75 v/v; Fig. 7d), methanol/phosphoric buffer (pH 5; 75/25 v/v; Fig. 7e), methanol/phosphoric buffer (pH 9; 75/25 v/v; Fig. 7f), ethanolic (Fig. 8a), ethanol/water (75/25 v/v; Fig. 8b), ethanol/water (50/50 v/v; Fig. 8c), and ethanol/water (25/75 v/v; Fig. 8d) extracts from chamomile, all heated under reflux.

The presented results show that the increase of chamomile extraction time in all the applied extractants leads to the increase of umbelliferone transformation degree to methyl or ethyl ester of *trans*-umbellic acid (transformation product **3** or **4**). The same effect for these compounds is observed in the artificial systems—their concentration grows with heating time increase in all umbelliferone alcoholic solutions (see Figs. 2, 3). For umbellic acid (no **2**), the second transformation product formed during umbelliferone extraction from chamomile, its concentration changes in ethanolic extractants are directly proportional to the extraction time (see Fig. 8). The concentration change of this umbelliferone derivative in methanolic chamomile extracts is less unequivocal (see Fig. 7). However, it can result from:

- more acidic character of methanol, in relation to ethanol, which favors the transformation of umbellic acid to its methyl ester and/or
- the influence of chamomile matrix components inhibiting/catalyzing this reaction.

The accidental error in quantitative analysis of umbellic acid cannot be excluded.

**Fig. 9:**
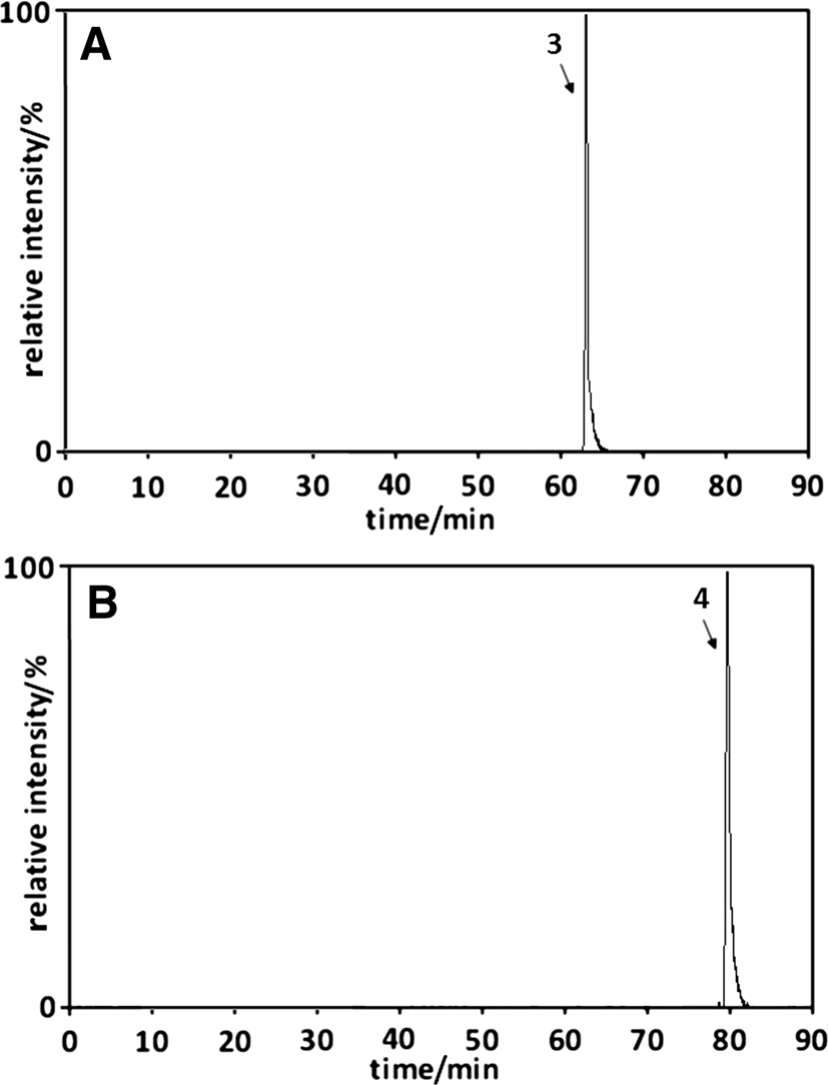
The SIM chromatograms of methanol/water (**a**; 75/25 v/v) and ethanol/water (**b**; 75/25 v/v) extracts from cinnamon bark, all heated under reflux for 2 h. Peak numbers correspond to compound numbers reported in Supplementary Scheme 1

For additional confirmation of umbelliferone transformation probability during food processing, further studies were conducted using cinnamon—plant material used as a seasoning for food or as a component of ready-to-eat foods. Figure 9 presents SIM chromatograms of methanol/water (75/25 v/v; Fig. 9a) and ethanol/water (75/25 v/v; Fig. 9b) extracts from cinnamon bark, all heated under reflux for 2 h.

The presented chromatograms show that each of the prepared extracts contains one umbelliferone derivative—*trans*-methyl ester of umbellic acid (peak **3**) and *trans*-ethyl ester of umbellic acid (peak **4**) in the case of methanolic and ethanolic extract, respectively. The absence of other derivatives found in the alcoholic–aqueous solutions of the umbelliferone standard and camomile extracts prepared in an analogous way may result from:

- their low concentration or
- the influence of the plant matrix components on the umbelliferone transformation process during the compound extraction from cinnamon bark.

## Conclusions

As known from literature [11, 26], umbelliferone can be transformed to hydroxylated (esculetin), glucosylated (skimmin), and methylated (herniarin) derivatives in the enzymatic degradation process. The presented paper shows that this compound is also chemical and thermal unstable. Many compounds can be formed from umbelliferone during its simulated and real extraction from food and plants by methanol, ethanol, buffers, and buffered or non-buffered alcohol/water mixtures. Except umbellic acid, none of the following have been reported yet:

4,7-dihydroxy-3,4-dihydro-2*H*-chromen-2-one;

3,7-dihydroxy-3,4-dihydro-2*H*-chromen-2-one;

methyl (2*E*)-3-(2,4-dihydroxyphenyl)prop-2-enoate;

ethyl (2*E*)-3-(2,4-dihydroxyphenyl)prop-2-enoate;

(2*E*)-3-[2-(acetyloxy)-4-hydroxyphenyl]prop-2-enoic acid;

(2*E*)-3-(2-amino-4-hydroxyphenyl)prop-2-enoic acid).

The amount of each formed component depends not only on extraction/heating time, alcohol concentration and extractant pH, but also on matrix components of the plant from which umbelliferone is extracted.

Umbelliferone instability should draw the attention of the manufacturers whose food, cosmetic, and health-promoting products contain this compound because the bioactivity of umbelliferone derivatives formed during the production process or preparations storage is still unknown. Moreover, the identified umbelliferone derivatives can be mistakenly treated as new components, not naturally present in the examined plant. Hence, the results may be important also for scientists studying the metabolism and/or transformation pathways of biologically active compounds such as umbelliferone in several botanical families [27, 28].

Therefore, the presented results are important not only in an industrial, but also in an analytical context.

## Experimental

The *Matricaria chamomilla* L. flowers, cultivated in eastern Poland, were air-dried and stored at + 4 °C. Ground and dried cinnamon bark was purchased from one of the local herbal shops. Sodium phosphate, phosphoric acid, ammonium chloride, ammonium hydroxide, sodium acetate, acetic acid, methanol, ethanol (all of analytical grade), and acetonitrile (HPLC) were purchased from the Polish Chemical Plant POCh (Gliwice, Poland). Umbelliferone standard (99%), umbellic acid standard (97%), and formic acid (98–100%) were purchased from Sigma-Aldrich (Seelze, Germany). Water was purified on the Milli-Q system from Millipore (Millipore, Bedford, MA, USA).

### Investigations of the umbelliferone transformation process during its extraction

The investigations of the umbelliferone transformation process were performed by heating under reflux its solutions in methanol or ethanol or water or buffer or alcohol/water or methanol/buffer mixtures. The alcohol/water solutions of umbelliferone contained 25 or 50 or 75% v/v of alcohol, whereas alcohol concentration in its methanol/buffer solutions was 75% v/v. Phosphate, ammonia and acetate buffers (pH 5 or 9) were used in the experiments. The glass equipment for the experiments consisted of a boiling flask (100 cm^3^) and a small condenser. The heated umbelliferone solutions contained 10 mg of the compound in 50 cm^3^ of a given solvent. To obtain a similar umbelliferone concentration in the chamomile flowers and cinnamon bark suspension in 50 cm^3^ of a given solvent, 2 or 4 g of the plant material, respectively, was used. Individual solutions and plant suspensions were heated for 1, 2 or 3 h at the boiling point of the used extractant. Subsequently, each obtained solution and supernatant isolated by centrifuging the plant suspension for 10 min at 13,000 rpm was subjected to LC–MS–PDA analysis.

### Investigations of umbellic acid cyclization process during the heating of its solution

The investigations of the umbellic acid cyclization process were performed in the same way and in the same equipment as those applied in investigations of the umbelliferone transformation process. In this case, three extractants containing 75% of alcohol were used: MeOH/phosphate buffer, pH 5; MeOH/phosphate buffer, pH 9; and EtOH/water. The influence of heating time (2, 4, 6, 8 or 10 h of heating at the boiling point of the used extractant) on umbellic acid cyclization process was studied.

### Estimation of umbelliferone concentration in chamomile flowers and cinnamon bark

The exactly weighed portion (0.3 g) of the ground plant material was ultrasonicated with 50 cm^3^ of methanol/water (75/25 v/v) mixture for 1 h at 40 °C. Subsequently, the supernatant isolated by centrifuging for 10 min at 13,000 rpm was subjected to LC–MS–PDA analysis.

### HPLC measurements

The chromatographic measurements were performed on an LC/MS system consisting of a UHPLC chromatograph (UltiMate 3000, Dionex, Sunnyvale, CA, USA), a linear trap quadrupole-Orbitrap mass spectrometer (LTQ-Orbitrap Velos from Thermo Fisher Scientific, San Jose, CA) and an ESI source. A Gemini C18 column (4.6 × 100 mm, 3 μm; Phenomenex, USA) was employed for chromatographic separation performed using gradient elution (Phenomenex pre-column, 4 × 3 mm, 3 μm, was used for column protection). Mobile phase A was 25 mM formic acid in water; mobile phase B was 25 mM formic acid in acetonitrile. The gradient program started at 5% B, increasing to 35% for 100 min, then 35% B to 95% B for 15 min, and ended with isocratic elution followed (95% B) for 10 min. The total run time was 125 min at the mobile phase flow rate 0.4 cm^3^ min^−1^.

In the course of each run, PDA spectra in the range 190–600 nm and MS spectra in the range of *m/z* = 100–2000 were collected continuously. In all the umbelliferone solutions and chamomile flower extracts, the SIM function was used to better visualize the chromatographic separation and to remove the signal connected with the umbelliferone, plant matrix, and buffer components. In the case of umbelliferone derivatives, the time periods and monitored ions were as follows: 0–30 min (*m/z* 178), 30–33 min (*m/z* 179), 33–38 min (*m/z* 179), 45–47 min (*m/z* 179), 47–50 min (*m/z* 179), 50–64 (*m/z* 193), 64–75 min (*m/z* 221), and 75–100 min (*m/z* 207); in the case of umbellic acid: 30–33 min (*m/z* 179), 33–38 min (*m/z* 179), 38–45 min (*m/z* 161).

The ESI was operated in negative polarity modes under the following specific conditions: spray voltage, 3.5 kV; sheath gas, 40 arbitrary units; auxiliary gas, 10 arbitrary units; sweep gas, 10 arbitrary units; and capillary temperature, 320 °C. Nitrogen (> 99.98%) was employed as sheath, auxiliary, and sweep gas. The scan cycle used a full-scan event at the resolution of 60,000.

To identify umbelliferone and umbellic acid derivatives (the products of its hydrolysis, transformation and oxidation), the function of secondary (MS^2^) ion fragmentation was applied. The collision energy for each examined compound was chosen individually. For confirmation, HRMS analysis was additionally performed.

Due to the lack of standards of umbelliferone derivatives, their amounts were estimated by relating their chromatographic responses to the calibration curve for umbelliferone (the concentration range 0.01–180 µg cm^−3^, *R*^2^ = 0.9998).

The calibration curve for umbelliferone was used for estimating the amounts of the following umbelliferone derivatives:

- 4,7-dihydroxy-3,4-dihydro-2*H*-chromen-2-one (**1**);
- 3,7-dihydroxy-3,4-dihydro-2*H*-chromen-2-one (**1**′);
- (2*E*)-3-(2,4-dihydroxyphenyl)prop-2-enoic acid (**2**);
- (2*Z*)-3-(2,4-dihydroxyphenyl)prop-2-enoic acid (**2′**);
- methyl (2*E*)-3-(2,4-dihydroxyphenyl)prop-2-enoate (**3**);
- ethyl (2*E*)-3-(2,4-dihydroxyphenyl)prop-2-enoate (**4**);
- (2*E*)-3-[2-(acetyloxy)-4-hydroxyphenyl]prop-2-enoic acid (**5**);
- (2*E*)-3-(2-amino-4-hydroxyphenyl)prop-2-enoic acid (**6**).

### Statistical analysis

All the results are presented as the mean of three independent measurements (*n* = 3). Differences in the concentration of the formed umbelliferone derivatives were compared using variance analysis (ANOVA, *p *= 0.05). Differences in the studied groups were considered significant for *p* values lower than 0.05 and *F* values higher than 5.14. Variance analysis revealed statistically significant differences for the tested groups.

## Electronic supplementary material

Below is the link to the electronic supplementary material.

Supplementary material 1 (DOCX 44 kb)
