# Supplementary material for: Umbelliferone instability during an analysis involving its extraction process
Source: Monatsh Chem. 2018 Jun 28;149(8):1327–40. doi: 10.1007/s00706-018-2188-9 (PMC6060953; doi:10.1007/s00706-018-2188-9)
Supplement: Supplementary file 1 — Supplementary material 1 (DOCX 44 kb) [file 706_2018_2188_MOESM1_ESM.docx]

**Umbelliferone instability during its analysis involving extraction process**

**Andrzej L. Dawidowicz^1^ ● Katarzyna Bernacik^1^ ● Rafał Typek^1^**

**Supplementary material**

**Scheme S1.** Molecular structures of umbelliferone transformation products identified in examined systems: (**1**) 4,7-dihydroxy-3,4-dihydro-2*H*-chromen-2-one; (**1**’) 3,7-dihydroxy-3,4-dihydro-2*H*-chromen-2-one; (**2**) (2*E*)-3-(2,4-dihydroxyphenyl)prop-2-enoic acid; (**2’**) (2*Z*)-3-(2,4-dihydroxyphenyl)prop-2-enoic acid; (**3**) methyl (2*E*)-3-(2,4-dihydroxyphenyl)prop-2-enoate; (**4**) ethyl (2*E*)-3-(2,4-dihydroxyphenyl)prop-2-enoate; (**5**) (2*E*)-3-[2-(acetyloxy)-4-hydroxyphenyl]prop-2-enoic acid; (**6**) (2*E*)-3-(2-amino-4-hydroxyphenyl)prop-2-enoic acid.
